# Positive direct antiglobulin test in post-artesunate delayed haemolysis: more than a coincidence?

**DOI:** 10.1186/s12936-019-2762-6

**Published:** 2019-04-08

**Authors:** Daniel Camprubí, Arturo Pereira, Natalia Rodriguez-Valero, Alex Almuedo, Rosauro Varo, Climent Casals-Pascual, Quique Bassat, Denis Malvy, Jose Muñoz

**Affiliations:** 1 ISGlobal, Hospital Clínic - Universitat de Barcelona, Barcelona, Spain; 2 Hematology Department, Hospital Clínic de Barcelona, Barcelona, Spain; 3 Internal Medicine Department, Hospital General de Granollers, Barcelona, Spain; 4 Microbiology Department, Hospital Clínic de Barcelona, Barcelona, Spain; 5 ICREA, Pg. Lluís Companys 23, 08010 Barcelona, Spain; 6 Centro de Investigaçao em Saúde de Manhiça (CISM), Maputo, Mozambique; 7 Pediatric Infectious Diseases Unit, Pediatrics Department, Hospital Sant Joan de Déu (University of Barcelona), Barcelona, Spain; 8 Travel Clinics and Tropical Diseases Unit, University Hospital Center of Bordeaux, Bordeaux, France

**Keywords:** Artesunate, PADH, Haemolytic anaemia, Direct antiglobulin test, Corticosteroids, Malaria

## Abstract

**Background:**

Delayed haemolysis is a frequent adverse event after treatment with artesunate (AS). Removing once-infected “pitted” erythrocytes by the spleen is the most accepted mechanism of haemolysis in these cases. However, an increasing number of cases with positive direct antiglobulin test (DAT) haemolysis after AS have been reported.

**Methods:**

All malaria cases seen at Hospital Clinic of Barcelona between 2015 and 2017 were retrospectively reviewed. Clinical, parasitological and laboratory data from patients treated with intravenous artesunate—specifically looking for delayed haemolysis and DAT—was collected.

**Results:**

Among the 36 severe malaria patients treated with artesunate at the hospital, 10 (27.8%) developed post-artesunate delayed haemolysis. Out of these, DAT was performed in six, being positive in four of them (at least 40%). DAT was positive only for complement—without IgG—suggesting drug-dependent immune-haemolytic anaemia of the immune-complex type. Three of the four patients were treated with corticosteroids and two also received blood transfusion, with a complete recovery.

**Conclusions:**

Drug-induced auto-immune phenomena in post-artesunate delayed haemolysis may be underreported and must be considered. The role of corticosteroids should be reassessed.

## Background

Intravenous artesunate (AS) has been established as the first-line treatment for *Plasmodium falciparum* severe malaria after demonstrating its superiority over quinine, in terms of reduction of mortality and neurological sequelae, in multicentre randomized clinical trials, with no serious drug-related adverse events initially reported [1–3]. However, as a consequence of the generalized use of AS, the post-artesunate delayed haemolytic anaemia is increasingly been recognized and reported, even following the administration of oral artemisinin-based combination therapy (ACT) [3, 4].

In spite of conflicting case-definitions [5, 6], post-artesunate delayed haemolysis (PADH) pattern has generally been defined as the presence of haemolysis with a > 10% decrease in haemoglobin level or a > 10% rise in LDH concentrations occurring more than 8 days after initiation of treatment [5]. PADH is estimated to occur in about 15–30% of malarial patients treated with AS, mainly affecting non-immune patients (presumably patients with no immunity to malaria) [7–9]. Although the pathophysiology of PADH remains unclear, splenic “pitting” of once-infected erythrocytes has been postulated as the most plausible mechanism of haemolysis [5–9]. Unlike other anti-malarial agents, artemisinins turn intraerythrocytic trophozoites into non-viable pyknotic forms which are removed by the spleen. It is supposed that the decreased lifespan of the resulting once-infected erythrocytes would lead to haemolytic events between 7 and 28 days after anti-malarial treatment was started [5–9]. Remarkably, although only a handful of cases with positive direct antiglobulin test (DAT) after AS has been described, they are increasingly being reported [10–16]. This fact raises the possibility of drug-induced autoimmune mechanisms contributing to PADH in some patients.

The objective of this report was to review the clinical and immunohaematological characteristics of patients with positive DAT in the context of PADH.

## Methods

All the 83 consecutive patients diagnosed with malaria attended at Hospital Clínic of Barcelona, Spain, between January 2015 and December 2017, were retrospectively reviewed. Thirty-nine per cent of them were migrants or visiting friends and relatives (VFR) and the remaining 61% were tourists or business travellers. Of these, specific clinical, parasitological and laboratory data was collected from the 36 patients diagnosed with severe malaria and treated with AS. Severe malaria was defined based on World Health Organization (WHO) malaria severity criteria [17]. PADH was defined as > 10% decrease in haemoglobin levels or > 10% rise in LDH concentrations occurring between the 8th and the 28th day after the first dose of AS, in patients with evidence of other parameters of haemolysis, such as hyperbilirubinaemia or decrease of haptoglobin in plasma. Hyperbilirubinaemia was defined as any increase above the normal limits with a previous normal determination or an increase > 10% if the previous determination was high. Decrease in plasma haptoglobin was defined as any decrease below normal limits with a previous normal determination.

The direct antiglobulin test (DAT), also known as direct Coombs test, was performed by the tube method with polyspecific antiglobulin (Anti-Human Globulin Mono-Type, Grifols Diagnostics AG, Düdingen, Switzerland) and monospecific anti-IgG and anti-C3d/C3c (both from Sanquin, Amsterdam, The Netherlands).

## Results

PADH was diagnosed in 10 of the 36 (27.8%) malaria patients treated with intravenous AS. Median age of the affected patients was 45 years (interquartile range (IQR): 33.25–54.25 years) with 80% being males. All patients lived in non-endemic areas and acquired the infection through travelling to malaria-endemic areas. No patients were considered semi-immune by the study investigators [18].

DAT was positive for complement and negative for IgG in four of the six *Plasmodium falciparum* malaria cases in which it was performed (see below), which represents at least 40% (4/10) of patients with PADH and 11.1% (4/36) of patients treated with AS. No IgG antibodies were found in plasma, and a cold agglutinin with limited thermal range was detected in one of these four patients. Three of the four patients with positive DAT were admitted to hospital and treated with high doses of corticosteroids, and two of them also required blood transfusion. All patients successfully recovered from the haemolysis and no relapses occurred. Due to the mildness of the anaemia, the remaining patient did not require any treatment. Interestingly, the four patients with positive DAT had initially presented with higher parasitaemia than the remaining six PADH patients (26.8%, IQR 16.9–36.8 vs. 5.65%, IQR 4.5–8.0; p = 0.019).

## Case descriptions

Cases with Coombs positive haemolytic anaemia after AS are described below.

### Patient 1

A 55-year-old European traveler presented to the hospital with a 2-day course of fever after travelling throughout Zambia, Malawi, Mozambique and South Africa without taking anti-malarial prophylaxis. A blood smear detected *P. falciparum* (parasitaemia 14.8%). The patient was diagnosed with severe malaria based on hyperparasitaemia and treatment with intravenous AS (2.4 mg/kg) was started. However, in the blood smear performed 8 h after AS initiation parasitaemia increased to 28.6%. In spite of not fulfilling the WHO criteria of AS resistance, treatment was changed to intravenous quinine (10 mg/kg/8 h after a loading dose of 20 mg/kg) and doxycycline (100 mg/12 h), under the clinical suspicion of decreased susceptibility of *P. falciparum* to AS. Blood smear performed 72 h after starting this treatment was negative. Quinine was interrupted after 5 doses due to ototoxicity and treatment was completed with atovaquone/proguanil for 3 more days.

Eight days after being discharged (and 14 days after the first dose of AS) the patient was admitted again for a 4-day course of fever, jaundice and dark urine. Two consecutive blood smears and a *Plasmodium* molecular screening by polymerase chain reaction (PCR) were negative. Laboratory tests showed haemolytic anaemia with hyperbilirubinaemia, elevation of LDH and undetectable haptoglobin. Haemoglobin levels decreased to 5.1 g/dL and DAT was positive only for the C3 fraction. Other causes of haemolytic anaemia were ruled out: no schistocytes were observed on the blood extension, glucose-6-phosphate dehydrogenase (G6PDH) deficiency and presence of antinuclear antibodies were discarded, as were other infectious aetiologies, such as common viral and bacterial infections. Treatment with corticosteroids with a loading dose of 250 mg of methylprednisolone and 1 mg/kg/day of prednisone was started. Transfusion of 4 erythrocyte concentrates was also required. The patient presented good clinical evolution allowing the discontinuation of corticosteroids after 2 months. Finally, the initial suspicion of AS resistance could not be proven by mutations on *kelch13*-propeller domain (*k13*).

### Patient 2

A 55-year-old splenectomized European traveller was admitted to the intensive care unit because of severe *P. falciparum* malaria 11 days after returning from Sierra Leone. He did not properly take the anti-malarial chemoprophylaxis with atovaquone/proguanil as prescribed. The patient was diagnosed with severe malaria due to hyperparasitaemia (4.7%), acute kidney injury and hyperlactacidaemia, that prompted a rapid initiation of intravenous AS (2.4 mg/kg). Although clinical and analytical improvement were observed, malaria blood smears conducted at 12 and 24 h after AS initiation showed a steady increase in parasitaemia up to 8.7%. Under the suspicion of AS resistance, treatment was changed to intravenous quinine (10 mg/kg/8 h) and doxycycline (100 mg/12 h) after the 4th dose of AS. Ten days after admission, the patient was discharged asymptomatic and in good clinical condition. Sequencing of *k13* showed no molecular markers associated to artemisinin resistance. A careful re-evaluation of malaria smears showed pyknotic non-viable forms due to the patient’s inability to remove erythrocytes from the bloodstream by “pitting”. A blood smear on day 28 was finally negative. During follow-up, microcytic anaemia (haemoglobin nadir of 9.8 g/dL at day 20) with hyperbilirubinaemia, increased LDH and undetectable haptoglobin was observed. DAT was positive for complement and cold agglutinin was detected in plasma. Given the mildness of the anaemia and the absence of symptoms, no treatment was required. A complete recovery was confirmed by laboratory tests after 2 weeks.

### Patient 3

Eight days after returning from Namibia, Botswana, Zimbabwe, Malawi and Mozambique, a 40-year-old European traveller was admitted to the hospital for a 6-day course of fever and chills. He did not take anti-malarial prophylaxis. Diagnosis of severe *Plasmodium falciparum* malaria based on hyperparasitaemia (45%), acute kidney injury and respiratory distress was made. The patient was admitted to the intensive care unit under mechanical ventilation, renal replacement therapy and 2.4 mg/kg of intravenous AS. After 5 doses a blood smear was negative. Anti-malarial treatment was completed with intravenous quinine and doxycycline for 5 more days due to oral intolerance. Despite the good parasitological response, renal disfunction progressed requiring renal replacement therapy. Thirteen days after AS initiation anaemia with haemolytic pattern was detected, with a nadir haemoglobin level of 6.7 g/dL at day 17. Glucose-6-phosphate dehydrogenase deficiency, schistocytes and other causes of haemolytic anaemia were ruled out and DAT resulted positive for the C3 fraction. The patient received a blood transfusion without anaemia improvement. Due to the kidney injury a renal biopsy was performed and reported as compatible with interstitial nephritis. Three pulses of 250 mg methylprednisolone were then prescribed. Regarding the anaemia, although a blood transfusion had previously been administered, haemoglobin concentration did not raise until corticosteroid treatment was initiated. The patient was finally discharged with a daily dose of 40 mg of prednisone and complete recovery was observed within the following month.

### Patient 4

A 49-year-old European man who frequently travelled to West Africa for business was admitted to the intensive care unit of the hospital after travelling to Liberia. He had not taken anti-malarial prophylaxis. The patient was diagnosed with severe *P. falciparum* malaria based on hyperparasitaemia (25%), acute kidney injury, respiratory distress and hyperbilirubinaemia. Following the hospital protocols, after the first intravenous AS dose, red blood cell exchange was performed. Subsequently, four more doses of intravenous AS were administered. The patient had an excellent clinical, analytical and parasitological evolution with a parasite clearance time of 70 h. Anti-malarial treatment was then completed with a 3-day course of dihydroartemisinin/piperaquine. Seven days after being discharged the patient had to be readmitted due to haemolytic anaemia, with a nadir haemoglobin level of 7.7 g/dL at day 16. DAT was positive for the C3d fraction of the complement and daily treatment with 100 mg methylprednisolone was started. Finally, a blood transfusion was performed after the DAT was negative. The patient was finally discharged with haemoglobin levels of 8.4 g/dL. Two weeks after, the patient remained in good clinical condition and the haemoglobin levels raised up to 9.6 g/dL.

## Discussion

Post-artesunate delayed haemolysis is increasingly being reported after anti-malarial treatment with artemisinins. In this series of 36 patients treated with parenteral AS, PADH occurred in over a fourth (27.8%) of patients treated with AS. Of these, DAT was positive in 4 of the 6 patients in whom the test was performed, which amounts to at least the 40% of patients with PADH. The true incidence of positive Coombs haemolysis after AS treatment is unknown. Since mechanical destruction of red blood cells (RBC) has been assumed as the main mechanism of haemolysis after AS, DAT has not been systematically performed and it has only been occasionally reported in most PADH case series. As a consequence, positive Coombs haemolytic anaemia after AS raises as a probable misdiagnosed entity.

Interestingly, in these 4 cases, DAT was positive only for complement—without IgG—as it was in two previously reported cases with specific information about the test [5, 10–16] (see Table 1). This pattern of DAT positivity is typical of the drug-dependent immune-haemolytic anaemia (IHA) of the immune-complex type [19], although it can also be seen in IHAs mediated by cold-agglutinins, biphasic haemolysins, and systemic immune-mediated disorders with strong complement activation, such some infectious events. There is little evidence about these immune delayed phenomena, but recent data suggest that post-malaria neurologic syndromes could also have an immunological mechanism [20]. Confirmation of drug-induced mechanism requires the demonstration of drug-dependent antibodies in the patient’s serum [10], which is a difficult task because of the intricacies of reproducing in vitro the in vivo conditions and because antibodies may recognize a metabolite instead of the original drug [20]. Positive DAT—with specific subclasses of IgG directed to parasite antigens—has been previously described in children from endemic countries with malaria and anaemia during the acute phase of the malaria infection [21, 22]. Despite that, the different pattern of DAT positivity might suggest a different pathophysiological mechanism of positive DAT delayed haemolysis, probably secondary to the use of AS [19].

**Table 1:** Summary of features of patients with immune-mediated post-artesunate delayed haemolysis (PADH)

Gender, age	Immunity	*Plasmodium* species	Parasitaemia (%)	Malaria severity criteria	Number of artesunate doses received	PCT (days)	PADH onset (day)	Hb nadir (g/dL)	Days to Hb nadir^a^
M 55	Non-immune	*P.f.*	28.6	Hyperparasitaemia	2	4	14	5.1	13
M 55	Non-immune	*P.f.*	8.7	Hyperparasitaemia, AKI, hyperlactaemia	4	20^c^	20	9.6	26
M 40	Non-immune	*P.f.*	45	Hyperparasitaemia, AKI, respiratory distress	5	3	9	6.1	12
M 49	Non-immune	*P.f.*	25	Hyperparasitaemia, AKI, respiratory distress, hyperbilirubinaemia	5	3	13	7.7	16
F 17	Semi-immune	*P.f.*	0.8	Shock, hyperlactaemia	3	3	14	4.6	16
M 43	Non-immune	*P.f.*	30	Hyperparasitaemia, prostration (psychomotor slow down), AKI, acidosis and hyperlactaemia	–	–	8	5.3	18
M 78	Non-immune	*P.f.*	27	Hyperparasitaemia, AKI	–	1.5	15	6.4	15
M 53	–	*P.f.*	34	Hyperparasitaemia, impaired consciousness, jaundice	2	4	–	6.9	20
F 44	–	*P.f.*	37	Hyperparasitaemia	3	4	–	6.1	15
F 59	–	*P.f.*	30	Hyperparasitaemia, haemoglobinuria, jaundice	1	10	–	6.9	13
M 54	Non-immune	*P.f.*	21	Hyperparasitaemia, AKI, shock, impaired consciousness, hyperbilirubinaemia	4	7	–	5.7	14
F 44	Non-immune	*P.f.*	37	Hyperparasitaemia, AKI, acidosis, purpura with thrombocytopenia	5	–	10	5.6	–
F 50	–	*Pf *+* P.vivax*	45	Hyperparasitaemia, AKI, acidosis, hyperlactaemia, hypotension, hyperbilirubinaemia	–	5	7	4.1	11
–	–	*P.f.*	–	–	–	–	–	–	–

Gender, age	Coombs	Treatment	Survival	Ref.	Country	Year	Frequency^b^
M 55	C3d+	Corticosteroids and blood transfusion	Yes	Our cases	Spain	2018	40% (4/10)
M 55	C3d+ and cryoagglutinins	None	Yes	Our cases	Spain	2018	40% (4/10)
M 40	C3d+	Corticosteroids and blood transfusion	Yes	Our cases	Spain	2018	40% (4/10)
M 49	C3d+	Corticosteroids and blood transfusion	Yes	Our cases	Spain	2018	40% (4/10)
F 17	IgG+ and C3d+ (positive drug-dependent antibodies test)	Corticosteroids	Yes	[10]	France	2014	100% (1/1)
M 43	C3d+	Corticosteroids	Yes	[11]	France	2018	100% (1/1)
M 78	Direct Coombs	Corticosteroids and blood transfusion	Yes	[12]	United Kingdom	2012	100% (1/1)
M 53	C3d+	None	Yes	[13]	Belgium/Netherlands	2012	50% (3/6)
F 44	IgG+ and C3d+	Corticosteroids and blood transfusion	Yes	[13]	Belgium/Netherlands	2012	50% (3/6)
F 59	IgG+ and IgM+	Corticosteroids and blood transfusion	Yes	[13]	Belgium/Netherlands	2012	50% (3/6)
M 54	IgG+ (anti-E specificity)	Blood transfusion	Yes	[14]	Germany	2012	33% (1/3)
F 44	No data	Blood transfusion	Yes	[15]	Spain	2017	25% (1/4)
F 50	IgG+	Corticosteroids and blood transfusion	Yes	[16]	India	2018	100% (1/1)
–	IgM+	–	–	[5]	France	2014	8% (1/13)

*AKI* acute kidney injury, *F* female, *Hb* haemoglobin, *M* male, *PCT* parasite clearance time, *P.f. Plasmodium falciparum, Ref* Reference

^a^Counted from artesunate initiation

^b^Frequency of immune-mediated PADH cases among the overall of PADH patients reported in each article

^c^Although non-viable pyknotic forms of *Plasmodium* were seen until day 20 after artesunate initiation, trophozoites could only be detected until day 6

Splenic “pitting” of parasites from infected erythrocytes, that has been related with initial parasitaemia, histidine-rich protein 2 (HRP2) and concentration of once-infected erythrocytes after treatment, has been proposed as the main mechanism for PADH [5]. These once-infected, “pitted” RBC become rigid spherocytes, and are removed from the circulation in subsequent passages through the spleen [5]. “Pitting” plays an important role in removing parasites from infected erythrocytes [23] and, in fact, PADH is more frequent in patients presenting with high parasitaemia or with a large number of residual spherocytes after effective treatment with AS [5]. However, given the mechanism of action of AS, “pitting” cannot explain all cases of haemolysis, as evidenced in the in splenectomized patients. Moreover, it is worth noticing that C3-coated RBC also become spherocytes before being removed from the circulation and might, therefore, be accounted for as pitted erythrocytes.

Furthermore, in all but one of the 4 patients with a positive DAT, PADH was severe enough as to require hospital admission and RBC transfusion. Treatment with corticosteroids in these 3 patients was followed by a rapid resolution of the haemolytic crisis, an outcome already observed in 6 of the 8 previously reported patients with PADH and positive DAT (see Table 1). However, it is important to be cautious before attributing therapeutic efficacy to corticosteroids in these cases. Indeed, both in PADH and drug-dependent IHA, the haemolytic process is self-limited once all the pitted erythrocytes have been removed and also when the drug has been cleared from the patient’s plasma.

This retrospective study has some limitations. On the one hand, DAT was only performed in some cases after the haemolysis appeared (6 of the 10 cases with PADH). As a consequence, DAT positivity due to malaria itself, although less probable, cannot be completely ruled out. The inclusion of a control group could have contributed to assess this problem. On the other hand, other causes of haemolysis such as drug-induced haemolysis due to different drugs such as beta-lactams, which were prescribed in the four patients, or quinine, which was given to 3 of the 4 patients, cannot be excluded. This is a hypothesis-generating study highlighting the importance of performing DAT when haemolysis after AS is detected.

## Conclusions

These cases, together with those previously published, highlight the possible contribution of autoimmune mechanisms to haemolysis due to artemisinins in selected cases and reopen the debate about the possible benefit of corticosteroids [10–13, 16]. Coombs positive haemolytic anaemia after AS is possibly underreported, due to the assumption of “pitting” as the mechanism of haemolysis. The presented results suggest that mechanical destruction of once-infected “pitted” erythrocytes is no longer the exclusive mechanism to explain haemolysis after AS. Consequently, DAT should be performed in all cases of suspected haemolysis during the follow-up of patients treated with AS in order to rule out immune-mediated haemolytic anaemia [24]. Further studies are necessary to clarify the role of immune-mediated mechanisms in PADH with positive DAT and the possible therapeutic efficacy of corticosteroids.

## Abbreviations

ACT: artemisinin-based combination therapy
AS: artesunate
DAT: direct antiglobulin test
G6PDH: glucose-6-phosphate dehydrogenase
HRP2: histidine-rich protein 2
IHA: immune-haemolytic anaemia
IQR: interquartile range
K13: *kelch* 13
LDH: lactate dehydrogenase
PADH: post-artesunate delayed haemolysis
PCR: polymerase chain reaction
RBC: red blood cells
WHO: World Health Organization
